# Cells collectively migrate during ammonium chemotaxis in *Chlamydomonas reinhardtii*

**DOI:** 10.1038/s41598-023-36818-6

**Published:** 2023-07-04

**Authors:** Gabela Nelson, Alexis Strain, Atsuko Isu, Alireza Rahnama, Ken-ichi Wakabayashi, Adam T. Melvin, Naohiro Kato

**Affiliations:** 1 Department of Biological Sciences, Louisiana State University, Baton Rouge, LA 70803 USA; 2 Laboratory for Chemistry and Life Science, Institute of Innovative Research, Tokyo Institute of Technology, Yokohama, Japan; 3 Cain Department of Chemical Engineering, Louisiana State University, Baton Rouge, LA 70803 USA; 4 School of Life Science and Technology, Tokyo Institute of Technology, Yokohama, Japan

**Keywords:** Cellular motility, Chemotaxis

## Abstract

The mechanisms governing chemotaxis in *Chlamydomonas reinhardtii* are largely unknown compared to those regulating phototaxis despite equal importance on the migratory response in the ciliated microalga. To study chemotaxis, we made a simple modification to a conventional Petri dish assay. Using the assay, a novel mechanism governing *Chlamydomonas* ammonium chemotaxis was revealed. First, we found that light exposure enhances the chemotactic response of wild-type *Chlamydomonas* strains, yet phototaxis-incompetent mutant strains, *eye3-2* and *ptx1*, exhibit normal chemotaxis. This suggests that *Chlamydomonas* transduces the light signal pathway in chemotaxis differently from that in phototaxis. Second, we found that *Chlamydomonas* collectively migrate during chemotaxis but not phototaxis. Collective migration during chemotaxis is not clearly observed when the assay is conducted in the dark. Third, the *Chlamydomonas* strain CC-124 carrying *agg1*^−^, the *AGGREGATE1* gene (*AGG1*) null mutation, exhibited a more robust collective migratory response than strains carrying the wild-type *AGG1* gene. The expression of a recombinant AGG1 protein in the CC-124 strain suppressed this collective migration during chemotaxis. Altogether, these findings suggest a unique mechanism; ammonium chemotaxis in *Chlamydomonas* is mainly driven by collective cell migration. Furthermore, it is proposed that collective migration is enhanced by light and suppressed by the AGG1 protein.

## Introduction

Taxis is the directed movement of organisms or cells in response to external stimuli. The essential physiological adaptation evolved among mobile organisms for survival and growth in dynamically changing environments. *Chlamydomonas reinhardtii* is a small (~ 10 µm) model organism for microalgae, possessing a pair of flagella, or cilia in a more specific term due to the microtubule composition^1^, on the anterior of an almost spherical cell. Microalgal cells migrate by beating the cilia symmetrically, like the movement of human arms when swimming the breaststroke. Because the angles of each cilia protruding from the anterior of the cell are slightly offset, the cell rotates during swimming^2^. Among the different types of taxes observed in *Chlamydomonas*, phototaxis is the most well-studied tactic behavior^3^. *Chlamydomonas* perceives light by an eyespot, a small (< 1 µm) structure where carotenoid granule layers are found between the inner and thylakoid membrane of the chloroplast^4^. When the eyespot is exposed to light from the surface of the cell, it reflects the light to the plasma membrane where channelrhodopsins 1 and 2 (ChR1 and ChR2) are localized*.* Excitation of ChR1 and ChR2 by the light then triggers a calcium influx. When the calcium concentration within the *trans*-cilium, located on the far side of the eyespot, is high, it beats stronger than the *cis*-cilium on the near side of the eyespot. This process causes the cell to steer towards the light source. This is deemed positive phototaxis. A mutant strain not having the eyespot (i.e., *eye3-2)*^5^ or a mutant strain with malfunctioning calcium flux in the cilia (i.e., *ptx1)*^6^ has been shown to be defective in phototaxis. When the intensity of the light increases (> 5 μmol photons·m^−2^ s^−1^), the cell migrates against the light source. This is deemed negative phototaxis^4^*.* When microalgae lack the *AGGREGATE1* gene, the cells, unless the cellular redox status is artificially alternated, migrate against the light source independent of the intensity of the light^7^. Although the localization of the AGGREGATE1 protein within the cells is known, the molecular function is not yet identified^7^.

*Chlamydomonas* also exhibits a directed tactic response to external gradients of selected chemicals (or chemotaxis). In 1992, Byrne et al. reported, using vegetative cells, that *Chlamydomonas* chemotaxis in response to an ammonium gradient (herein called ammonium chemotaxis) relies on the circadian rhythms that are grown with light:dark (12 h:12 h) cycle^8^. Ammonium chemotaxis is most active during the nighttime (dark cycle) and least active during the daytime (light cycle). The assay for this study was conducted in the dark for only 30 min. This study also reported, using [^14^C] methylammonium, that ammonium uptake is most active during the daytime and least active during the nighttime. They suggested that, in nature, *Chlamydomonas* may find a nutrient-rich environment during the nighttime and then wait until the sun rises to take up and assimilate ammonium using a light-dependent mechanism. However, unlike phototaxis, the mechanisms governing chemotaxis in *Chlamydomonas* are largely unknown at both the systems and molecular levels. A study by Ermilova et al. isolated a *Chlamydomonas* mutant strain that was defective in ammonium chemotaxis by DNA-insertional mutagenesis^9^. Additionally, Ermilova et al. isolated a *Chlamydomonas* mutant strain that suppresses ammonium chemotaxis with a potassium-channel inhibitor^10^. These studies showed that the mutant strains were deficient in high-affinity ammonium transport. Despite the extended work by Ermilova et al., none of the gene loci of these mutant strains were identified. Publications by other research groups concerning the genetic analysis in algal chemotaxis, including *Chlamydomonas,* are not available in scientific-publication databases. These limitations make it difficult to further elucidate the underlying mechanisms regulating *Chlamydomonas* chemotaxis based on the previously published literature. In 2009, Polin et al. found that *Chlamydomonas* swim in the dark stochastically by switching between synchronous and asynchronous beating between *cis*- and *trans*- cilia^11^. Because the stochastically swimming pattern resembles the “run-and-tumble” motion used for bacteria to sense and migrate towards a source during chemotaxis, the authors suggested a possible interplay of the stochastic switch of cilia beating in *Chlamydomonas* chemotaxis; however, no additional studies have been reported investigating this hypothesis.

Collective migration has been shown to occur during the directed migration of bacteria, animals, and mammalian cells in chemotaxis. Collective migration is defined as group movement in which individual cells or organisms (i.e., birds) migrate in concert with one another^12^. Collective cell migration promotes directional migration towards or against a stimulus^13,14^. For instance, bacterial aggregation prior to chemotaxis optimizes the search for nutrients^15^. Formation of an epithelial sheet provides strong, stable intercellular junctions in the tissue during wound healing^16^. Three types of mechanisms have been discovered in collective cell migration. The first type is used mainly by animal cells that utilize cell–cell interactions where these physical interactions between the cells via specialized molecules maintain cluster cohesion^17^. During initiation, individual cells migrate near the center of the population and then migrate towards the source as a group^18^. The second type is quorum sensing, used mainly by bacteria, where cells sensing a chemical gradient release extracellular signaling molecules to attract others^19^. The third type, also used mainly by bacteria, relies on individual cells responding to local nutrient levels based on their position in a group^14^. A cell consumes a nutrient that is locally available resulting in the establishment of a steep gradient of nutrients at the single-cell level which extends across the population. The gradient biases the random-walk motion in individual cells, making the population collectively migrate. An explanation of collective migration from a biophysics point of view can be found elsewhere^20^. It is currently unknown whether collective migration occurs during chemotaxis in microalgae, including *Chlamydomonas*.

One of the challenges associated with an incomplete understanding of the mechanisms governing *Chlamydomonas* chemotaxis is the assays used to study this behavior. Nearly all of the *Chlamydomonas* chemotaxis assays mentioned above were conducted using an apparatus optimized for bacterial chemotaxis in which light does not affect the tactic response^8,21,22^. In this method, cells are deposited into a culture dish that does not contain ammonium. Then two 3–5 mm capillary tubes, one filled with an ammonium solution and one with media lacking ammonium (control), are placed into the culture dish. The cells migrate into the tubes within 10–30 min, the tubes are then collected, and the number of cells in the tubes is counted and compared. A cell number ratio in the ammonium-containing tube compared to that in the control tube is used to calculate the chemotaxis index. In this assay, it is difficult to expose light in the tube and culture dish homogeneously, which can create a significant bias based on the light gradient. It is also difficult to monitor the patterns of migration within the tubes directly. This results in an inability to determine any change in the rate that the cells move into and out of the tube. We believe these factors make an analysis of the mechanisms regulating ammonium chemotaxis difficult. To address this limitation, new methods using microfluidic devices to study *Chlamydomonas* chemotaxis have been developed^23,24^. Microfluidics possess the superior capability to generate a well-defined, stable chemical gradient while allowing direct observation of cell migration with a microscope. These devices have the potential to change how *Chlamydomonas* chemotaxis is analyzed; however, these microfluidic devices were not designed for homogenous light exposure to the sample^23,24^. Moreover, microfluidic devices require specialized equipment to be produced, potentially limiting their utility in studying *Chlamydomonas* chemotaxis. We sought to develop an alternative method to study chemotaxis in *Chlamydomonas*. To this end, we developed a simple method that is easily accessible and repeated with minimal effort that overcomes the problems associated with the capillary method. This report describes the new assay and how it was used to discover new findings about ammonium chemotaxis in *Chlamydomonas*.

## Results

### Petri dish assay was established to analyze ammonium chemotaxis in *Chlamydomonas*

**Figure 1:**
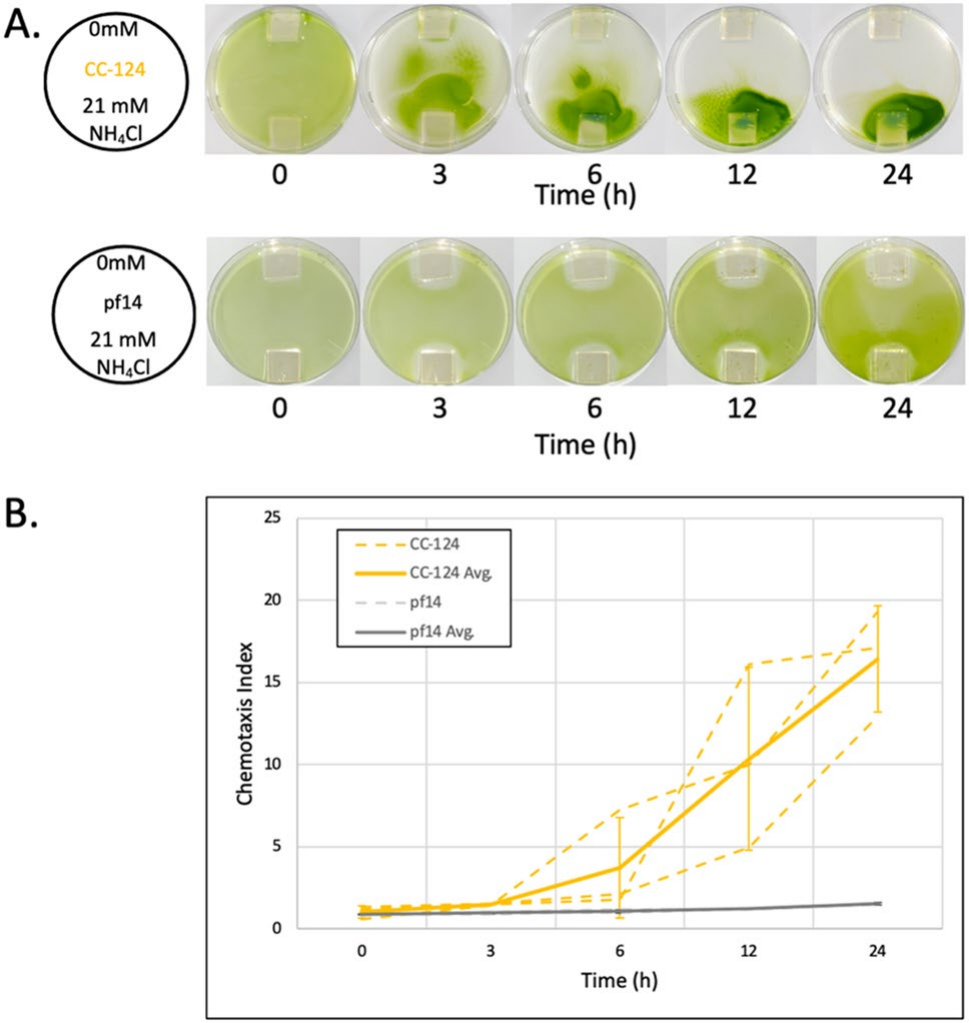
Petri dish assay and quantification for *Chlamydomonas* ammonium chemotaxis. (**A**) Photos show the directed cellular migration of a population of *Chlamydomonas* CC-124 over 24 h compared to the immobilized/paralyzed strain (*pf14*). Agarose blocks containing 0 mM and 21 mM NH_4_Cl were placed on the opposite sides (top and bottom, respectively, in the photos) in a Petri dish (diameter 100 mm). (**B**) Chemotactic Index (CI) was determined at 0, 3, 6, 12, and 24 h time points. Grey dotted lines connect the CI values at each time point. The results of three independent experiments for CC-124 and *pf14* were shown (orange and dark grey dashed lines, respectively). The solid orange line connects the average CI value of CC-124 at each time point of the three independent experiments (CC-124 Avg.). The dark grey solid line connects the average CI values of *pf14* at each time point of the three independent experiments (*pf14* Avg.). Error bars at each time point indicate the standard deviation of the three experiments in CC-124 and *pf14*, respectively.

Chemotaxis assays were conducted in a photo box to create homogenous light exposure to the top of a Petri dish where *Chlamydomonas* was placed in a medium lacking ammonium (Supplementary Figs. S1A,B). The cells duplicate at 14.3 ± 4.0 h (N = 3) with homogenous light and an ammonium supply in the dish. We used cultures harvested during their night cycle based on the previous report that finds ammonium chemotaxis would be most active during the nighttime in *Chlamydomonas*^8^. Agarose blocks containing either 0 mM NH_4_Cl (sink) or 21 mM NH_4_Cl (source) were positioned in the dish. Cells were homogeneously distributed across the dish at the start of experimentation when using the Petri Dish assay to assess the chemotactic response of *Chlamydomonas*; however, the cells were observed to begin migrating towards the source agarose (containing 21 mM NH_4_Cl) within 3 h (Fig. 1, Supplementary Movie S1). By 12 h, the migration towards the source agarose was clearly observed where the cells remained near the source agarose even after 24 h of experimentation.

To validate chemical gradient formation via chemical diffusion, we tracked changes in pH in a dish with a bromophenol blue solution, a pH-sensitive colorimetric dye (Supplementary Movie S1). At 3 h after the setup, the ammonium solution diffused through ~ 50% of the Petri dish and ultimately diffused across most of the dish by the 24 h time point. Additionally, a computational simulation was performed to model the mass transfer (Supplemental Fig. S2). The mass transfer of the tracer dye (bromophenol blue) was found to fit the known model for one-dimensional mass transfer following Fick’s Law (Eq. S2), validating the presence of a chemical gradient across the dish for the entire 24 h experiment.

These observations confirmed both the formation of an ammonium gradient in the dish for up to 24 h and the directed cellular migration towards the source. A linear correlation between color intensities and cellular densities was observed for densities of 3 × 10^4^ to 3 × 10^6^ cells/ml to allow for quantifying directed cellular migration (Supplementary Fig. S3). Changes in color intensity around the agarose blocks were measured to track changes in the spatially local density of *Chlamydomonas* cells. The migration of the algal population (defined by the Chemotactic Index or CI) was quantified as a ratio of the color intensity around the agarose containing 21 mM NH_4_Cl to the agarose containing 0 mM NH_4_Cl as defined by:

1 $$CI = \left[ {\frac{{{\text{Average}}\;{\text{intensity}}\;{\text{around}}\;{\text{source }}}}{{{\text{Average}}\;{\text{intesity}}\;{\text{around}}\;{\text{sink }}}}} \right]$$

In an experiment, when *Chlamydomonas* ammonium chemotaxis was assayed under homogeneous light, the cellular density around the source agarose block (containing NH_4_Cl) increased to a CI of 1.49 ± 0.04 at 3 h in three independent experiments. At 6 h, the CI increased to 3.72 ± 3.05 while by the 24 h time point, the CI reached 16.00 ± 3.23 (Fig. 1). Conversely, when the *pf14* mutant strain (paralyzed mutant due to lack of radial spokes in cilia, Supplementary Table S1) was assayed under homogeneous light, the CI was less than 1.22 ± 0.03 by 12 h and did not exceed 2.00 after 24 h of observation in three independent experiments (Fig. 1). This suggests that a CI value greater than or equal to 2.00 would represent a positive chemotactic response at any given time using the Petri dish assay regardless of the cell duplication time.

### Homogeneous light exposure enhances ammonium chemotaxis in *Chlamydomonas*

**Figure 2:**
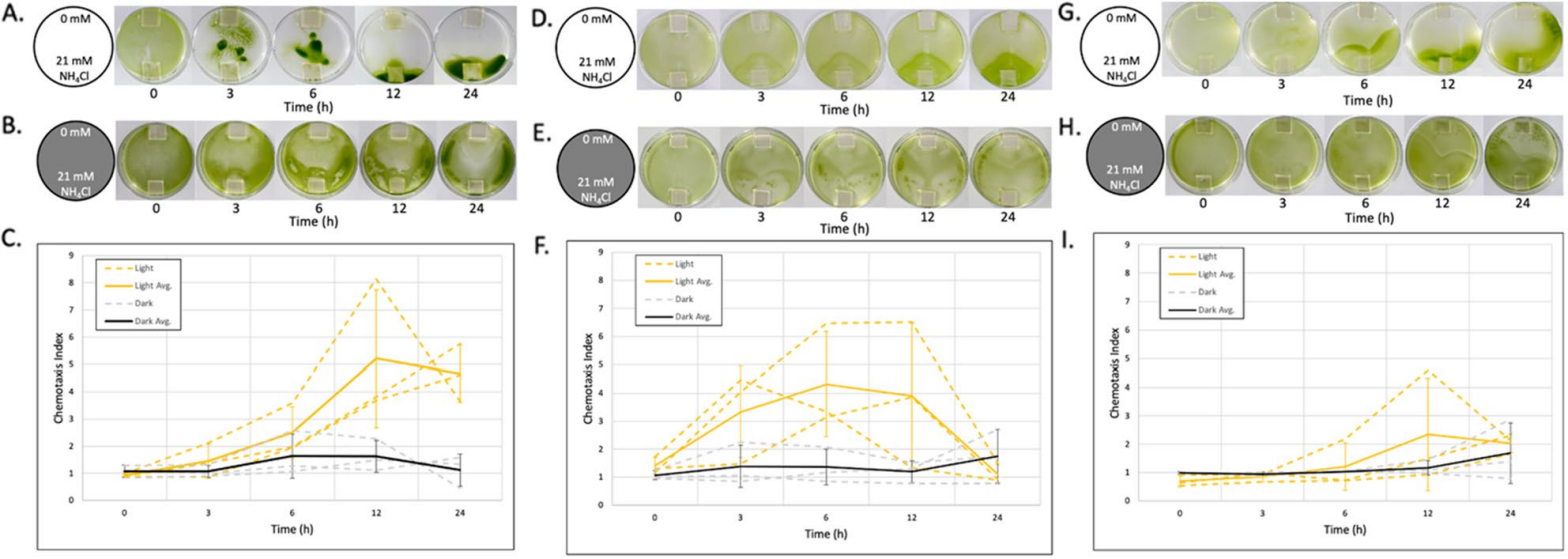
Ammonium chemotaxis of *Chlamydomonas* is enhanced by light across multiple strains. *Chlamydomonas* cells (**A**–**C**: CC-124, **D**–**F**: CC-125, and **G**–**I**: CC-4533) were exposed to chemical gradients under homogenous white light (Light) (**A**, **D**, and **G**) or in the dark (Dark) (**B**, **E**, and **H**). An agarose block containing 0 mM NH_4_Cl and 21 mM NH_4_Cl (respectively, top and bottom in the photos) was placed in the Petri dish. Photos were taken at 0, 3, 6, 12, and 24 h time points after setup. Photos shown above each graph represent algal migration in each experiment. (**C**, **F**, and **L**): Orange and grey dotted lines connect the chemotaxis index (CI) values at each time point. The results of three independent experiments in light (orange dotted line, Light) and dark (grey dotted line, Dark) were shown, respectively. The solid orange line connects the average CI value with light (Light Ave.) at each time point of the three independent experiments. The dark grey solid line connects the average CI values in the dark (Dark Avg.) at each time point of the three independent experiments. Error bars at each time point indicate the standard deviation of the three experiments.

Previous studies on the light dependency in ammonium chemotaxis in *Chlamydomonas* have been unclear and inconsistent among published reports^8,22,25^. To elucidate the role of light during ammonium chemotaxis, we first conducted experiments to clarify light dependency. In our assay, we used cultures 3–5 h into their night cycle (nighttime) that were grown in a 250 ml flask with 100 ml of medium containing acetate (TAP medium) for 3 or 4 days in a light:dark (12 h:12 h) cycle (mixotrophic culture). During the assay, the light (about 30 μmol photons·m^−2^ s^−1^) was continuously exposed to the top of the Petri dish for 24 h (Light), in which the culture medium filled the Petri dish about 2 mm in height. Alternatively, a second assay was conducted without exposing the culture to homogeneous light (Dark). We used the *Chlamydomonas* strains CC-124, CC-125, and CC-4533, which are often referred to as wild type in the scientific community studying *Chlamydomonas*, yet have been shown to exhibit genetic variations^7^ (Supplementary Table S1). The CC-124 strain carries the *AGGREGATE1* gene null mutation (hereafter, *agg1*^−^) that causes *Chlamydomonas* to migrate away from a light source (negative phototaxis)^7,26^. The other two strains, CC-125 and CC-4533, carry a wild-type *AGGREGATE 1* gene (hereafter, *AGG1*) and migrate towards or away from the light source depending on light intensity (neutral phototaxis). CC-4533 is a cell wall deficient strain (*cw15*) used for the insertional mutant library CLiP (Chlamydomonas Library Project) and widely used for studies in *Chlamydomonas* genetics^27^. Despite the variants exhibiting different genetic properties, all strains exhibited positive chemotaxis responses under homogeneous light exposure (Light, Fig. 2). In contrast, no strains showed a strong chemotactic response in the dark (Dark, Fig. 2).

Among the strains tested, CC-124 exhibited the most concentrated populations of cells near the source agarose block under homogeneous light exposure (Light) (Fig. 2A). To examine whether this strong collection of cells near the source agarose exhibited by CC-124 is due to the directed migration of the cells on the bottom of the Petri dish (i.e., cells adhere to and glide on the surface of the dish^28^) as the result of negative phototaxis away from the overhead light source in the z direction, we exposed the light from the bottom of the dish. We did not observe any differences in the chemotactic response of CC-124 between the top and bottom light exposures (Supplementary Fig. S4). We also found that *pf18*, the *Chlamydomonas* mutant strain that can glide but not swim^29^, is defective in ammonium chemotaxis (Supplementary Fig. S7), suggesting the most concentrated population of cells near the source agarose in CC-124 is not due to the migration of the cells being driven by gliding on the surface of the dish but due to swimming towards a specific location. We also observed that ammonium chemotaxis occurred similarly in both daytime and nighttime samples when the assay was conducted with continuous light (Supplementary Fig. S5). Furthermore, we discovered that ammonium chemotaxis rarely occurs when *Chlamydomonas* is cultured autotrophically and assayed in a medium not containing acetate (Supplementary Fig. S6). Based on these findings, we performed all remaining experiments, unless otherwise specified, using 10^6^ cells/ml of *Chlamydomonas* cultured with acetate (TAP medium) during the night cycle (nighttime), 3–5 h into the dark on the day:night (12 h:12 h) cycle.

### Ammonium chemotaxis occurs independently from the eyespot, calcium-dependent shift in ciliate, or ammonium uptake

Because we observed that homogeneous light exposure during the assay enhanced ammonium chemotaxis (Fig. 2), we sought to investigate if ammonium chemotaxis could occur in a mutant strain that lacks the eyespot structure (*eye3-2*, CC-4316) required for phototaxis^30^. We also studied a mutant strain deficient in a calcium-dependent shift in ciliate dominance required for phototaxis (*ptx1*, CC-2894)^6^. Both mutant strains exhibited a CI value greater than 2.00 after 6 h (Supplemental Fig. S7), indicating positive ammonium chemotaxis. These results suggested that ammonium chemotaxis does not require the same underlying mechanisms algae use during phototaxis. We also examined a mutant strain deficient in ammonium uptake (*amt4*^*−*^, CC-4042)^31^ to examine the possibility that a migratory response to the ammonium gradient was determined by cellular uptake of ammonium as has been shown to be the case in bacteria^14^. Similar to the other mutant strains, CC-4042 exhibited a CI value greater than 2.00 after 6 h (Supplemental Fig. S7), indicating positive ammonium chemotaxis. This result suggests that the cellular uptake of ammonium does not determine the chemotactic response towards the ammonium gradient.

### *Chlamydomonas* exhibits collective cell migration during ammonium chemotaxis

**Figure 3:**
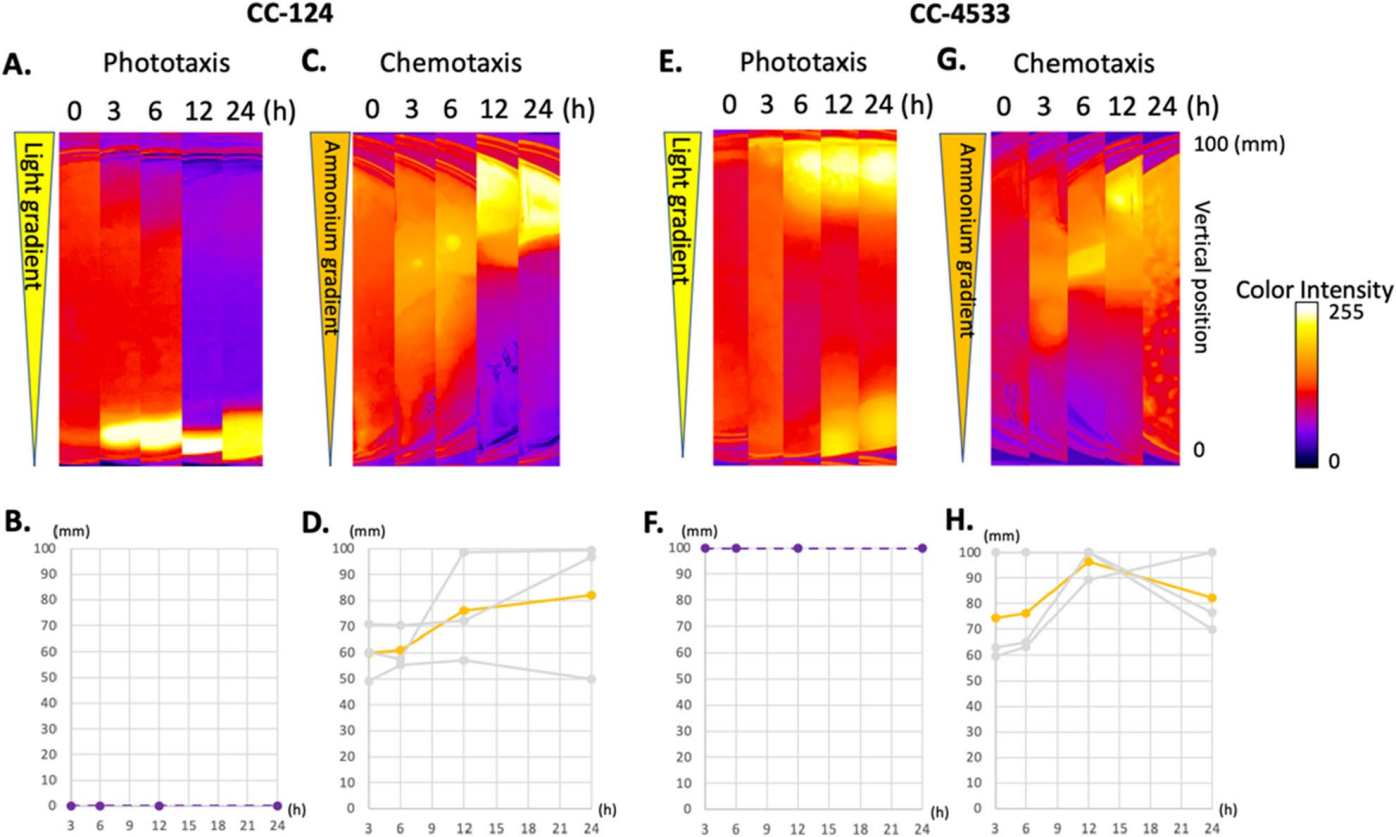
*Chlamydomonas* exhibit collective cell migration during ammonium chemotaxis. Phototaxis and chemotaxis of CC-124 and CC-4533. The cells were exposed to light gradients (**A**, **B**, **E**, **F**) or chemical gradients (**C**, **D**, **G**, **H**). The light gradient (~ 30 μmol photons·m^−2^·s^−1^ at the open side of the converted box) was formed from the top to the bottom of the Petri dish in the images (shown as an elongated triangle). The chemical gradient (21 mM to 0 mM NH_4_Cl) was formed from the top to the bottom of the Petri dish in the images (shown as an elongated triangle). After setup, the photos were taken at 0, 3, 6, 12, and 24 h time points. Changes in the local cell densities in the Petri dish for CC-124 and CC-4533 were shown as heat maps (**A**, **C**, **E**, **G**). Changes in the density center in the center strip of the dish were graphed with a function of time (**B**, **D**, **F**, **H**). The bottom and top rims of the dish in the photos were defined as 0 and 100 mm, respectively. Grey lines are the results of three independent experiments. Colored lines are the average of the three experiments. Notice that the density centers in three independent experiments in phototaxis are always 0 and 100 mm in CC-124 and CC-4533, respectively. On the other hand, the density centers in three independent experiments in chemotaxis are first observed near the center of the Petri dish.

All of the chemotaxis experiments performed using the Petri dish assay across the various *Chlamydomonas* strains identified that the coefficient of variability with respect to the CI across three independent experiments tended to be constantly high. One possible explanation is that some subpopulations of cells migrate collectively, hindering the continuous and steady accumulation of cells near the source agarose. To investigate the possibility of collective migration in ammonium chemotaxis, we compared migration patterns in a Petri dish during either ammonium chemotaxis or phototaxis. For the phototaxis assay, we placed a *Chlamydomonas* culture in a Petri dish with no agarose blocks resulting in no chemical gradient. The Petri dish was then covered with a cardboard box in which one side was cut off so that a light gradient was created over the Petri dish (Supplementary Fig. S8). *Chlamydomonas* prepared from the same culture were used for both phototaxis and chemotaxis assays. Two Petri dishes, one for the chemotaxis assay and the other for the phototaxis assay, were placed in the same photo box. To evaluate the migration pattern quantitatively, a pixel cluster in which the highest density of cells was detected in the Petri dish images was tracked with a function of time. Two strains, CC-124 (*agg1*^−^, negative phototaxis strain) and CC-4533 (*AGG1*, neutral phototaxis strain) were examined (Fig. 3). During phototaxis, an area of the highest density of *Chlamydomonas* CC-124 was detected near the edge of the Petri dish (the darker side of the light gradient) after 3 h. This high density of cells remained near the edge throughout the duration of the experiment. Similarly, an area with the highest density of *Chlamydomonas* CC-4533 was detected near the edge of the Petri dish (the brighter side of the light gradient) after 3 h. This high density of cells remained near the edge throughout the duration of the experiment; however, a small portion of the cells did accumulate near the negative side of the gradient at the 12 and 24 h time points. These findings suggest that the cells migrate individually and constantly towards or away from the light source during phototaxis.

During chemotaxis, an area with the highest density of CC-124 and CC-4533 was identified near the center of the Petri dish after 3 h. This area then moved towards the source agarose over time, with the highest density area reaching the ammonium source by 24 h. This migration behavior, where individual cells first migrate towards the center of the population and then migrate towards the source as a group, is a hallmark of collective migration often observed in animal cells utilizing specialized molecules to maintain cluster cohesion^18^. Accordingly, we concluded that *Chlamydomonas* collectively migrates during ammonium chemotaxis. We also concluded that collective migration occurs independently from the genomic status of the *AGG1* gene. To observe the collected migration at the microscopic level, we placed the Petri dish in which CC-124 was migrating towards an ammonium source on an inverted transmission microscope. While the movement of the cellular aggregate was observed, the movement of the cilia of aggregated cells was not clearly observed. The movement of the cellular aggregate appeared to occur by the activity of non-aggregated cells propelling the aggregate towards the ammonium source (Supplementary Movie S2).

### *Chlamydomonas* lacking the *AGG1* gene enhances ammonium chemotaxis by collective migration

**Figure 4:**
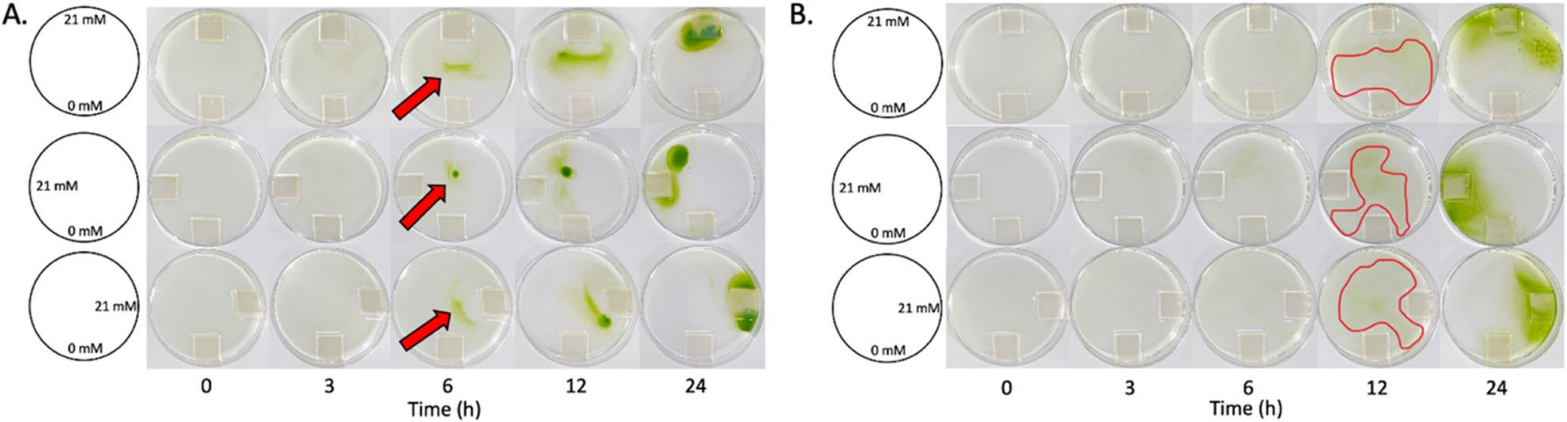
CC-124 (*agg*1^-^) but not CC-4533 (*AGG1*) exhibits clear collective migration during ammonium chemotaxis. *Chlamydomonas* with a low cell density (10^5^ cells/ml) were exposed to chemical gradients from three separate directions by placing source agarose blocks in different locations within the Petri dish. The top row has a chemical gradient from 0 mM NH_4_Cl at the bottom of the Petri dish to 21 mM NH_4_Cl at the top of the Petri dish in the photos. The second row has a chemical gradient from 0 mM NH_4_Cl at the bottom of the Petri dish to 21 mM NH_4_Cl at the left of the Petri dish in the photos. The third row has a chemical gradient from 0 mM NH_4_Cl at the bottom of the Petri dish to 21 mM NH_4_Cl at the right of the Petri dish in the photos. Photos were taken at the 0, 3, 6, 12, and 24 h time points for either CC-124 (**A**) and CC-4533 (**B**). The red arrows in (**A**) indicate collected CC-124 cells, whereas the highlighted red areas in (**B**) indicate collected CC-4533 cells.

Although collective migration was observed in both CC-124 and CC-4533 (Fig. 3), we observed the collective migration in CC-124 (*agg1*^−^) more clearly than in CC-4533 (*AGG1*) under homogeneous light exposure (Fig. 2). To investigate the effect of the *AGG1* gene in collective migration, we first conducted ammonium chemotaxis experiments with a low density of cells (10^5^ cells/ml) for both CC-124 and CC-4533 because the lower density allowed a clearer observation of the collective migration response compared to the standard density used in the Petri dish assay (10^6^ cells/ml) (Fig. 4).

**Figure 5:**
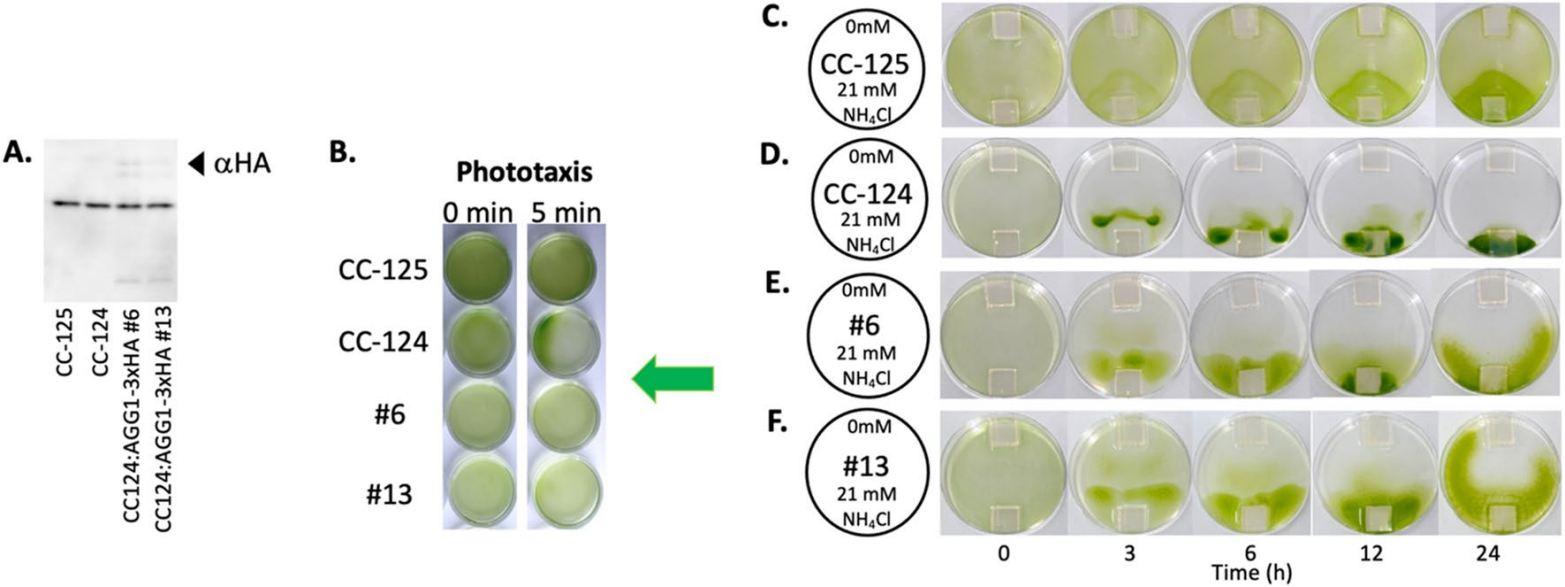
Expression of recombinant AGG1 protein in CC-124 suppresses collective migration during ammonium chemotaxis. (**A**) Expression of the recombinant AGG1 protein in the transgenic CC-124 was confirmed by western blotting. Protein extracts in the transgenic CC-124 lines #6 and #13 that express AGG1-3xHA^7^ were subjected to western blot analysis using an anti-HA antibody (αHA). The recombinant protein (estimated mass of 40.6 kDa) that appeared in the blot is indicated by an arrowhead. The original, unprocessed image of the blot was shown in Supplementary Fig. S11. (**B**) Transgenic CC-124 lines #6, and #13 were subjected to Wakabayashi’s phototaxis assay^32^. As controls, CC-124 (negative phototaxis) and CC-125 (neutral phototaxis) strains were also subjected to the assay. A green arrow indicates the direction of the light. (**C**–**F**) Photos show migration patterns of CC-125, CC-124, #6, and #13 over 24 h. In a Petri dish, agarose blocks containing 0 mM and 21 mM NH_4_Cl were placed on opposite sides (top and bottom, respectively, in the photos). Notice the reduction of collective migration in #6 and #13 compared to CC-124. Five-minute interval time-lapse movies are presented in Supplementary Movie S3.

To investigate a potential light-gradient bias against migration towards an ammonium source during the Petri dish assay, the source agarose was placed in three different locations in Petri dishes. Both strains (*agg1*^*-*^ and *AGG1*) show collected cells in the center of the Petri dish after 6 h (CC-124) or 12 h (CC-4533) of experimentation independent of the location of the source agarose; however, the collection of cells was more prominent in CC-124. The collected cells were then observed to migrate towards the source agarose over time after the initial clustering event. This result suggests that light-gradient bias does not exist against the collective migration of *Chlamydomonas* towards the ammonium source. It again further suggests that collective migration occurs independently from the *AGG1* gene status in the genome; however, the collective migratory response is more prominent in cells lacking the *AGG1* gene. In the experiments conducted in the dark, low-density CC-124 (10^5^/ml) migrated towards the source agarose, although clear collective migration was not visible. On the other hand, we could not identify the low-density CC-4533 migrating towards the ammonium source (Supplemental Fig. S9). These findings suggest that lacking the *AGG1* gene enhances ammonium chemotaxis in the dark. To examine the hypothesis that lacking the *AGG1* gene enhances collective migration during ammonium chemotaxis, the Petri dish assay was conducted with transgenic CC-124 in which the recombinant AGG1 protein was expressed through the *agg1*promoter::*AGG1*-*3xHA* (Human influenza hemagglutinin) gene expression cassette^7^. The two independent transgenic strains, named #6 and #13, showed reduced collective migration (Fig. 5, Supplementary Movie S3), supporting the hypothesis that cells lacking the *AGG1* gene exhibited enhanced collective cell migration during ammonium chemotaxis.

## Discussion

### A novel Petri dish assay to study chemotaxis reveals different tactic responses to an ammonium gradient, compared to a light gradient, in *Chlamydomonas*

A simple method was developed to study *Chlamydomonas* ammonium chemotaxis in a Petri dish (Fig. 1). This method was also demonstrated to be able to study *Chlamydomonas* phototaxis (Fig. 3). The materials required for the assay are readily available at local or online stores. It is easy to set up and manipulate light and chemical gradient conditions. Another advantage of this new assay is that it is spatially visible while still able to expose cells to homogenous light in the Petri dishes allowing for the ability to visualize cellular behavior such as collective migration. Prior studies utilized capillary tubes or microfluidic devices to analyze *Chlamydomonas* chemotaxis^21,23^; however, in these methods, it is difficult to observe the migration of the entire *Chlamydomonas* population. We observed collective migration in the Petri dish assay, which was not observed during phototaxis in *Chlamydomonas* (Figs. 3, 4). One disadvantage of this new approach, which was shared with both the capillary tube method and the microfluidic device, is that a steady-state chemical gradient across the entire dish was not established prior to the initiation of the migration studies. This non-steady concentration gradient could potentially explain the observed high coefficient of variation of the fold changes in the chemotactic index as the cells are responding to the developing gradient (Fig. 2). This suggests that the Petri dish assay may not be suitable for detecting small changes in the chemical gradient at the single-cell level during cellular migration caused by genetic variants or environmental conditions.

We observed that *Chlamydomonas* was very sensitive to a light gradient in the Petri dish assay. Unless homogenous light was exposed during the assay, *Chlamydomonas* were found to be heavily biased towards the light gradient. This suggests that the phototactic response may be prioritized over the chemotactic response in *Chlamydomonas*. Another finding is that clear detection of *Chlamydomonas* migration to an ammonium source agarose block takes a longer time (6–12 h) compared to that towards a light source (< 3 h) in the assay using a 100 mm diameter Petri dish. This slow migratory response partially agrees with the results of the studies using a microfluidic experiment. This study found that it took on average 3 h for *Chlamydomonas* to cross a 4.6 mm long channel (155 µm wide) during an ammonium chemotaxis^23^. This calculates an average migration speed of ~ 0.3 µm s^−1^ during chemotaxis in the microfluidic device. Conversely, a previous study estimates that an average migration speed during phototaxis could be as fast as 78 µm s^−1^^33^. In our Petri dish assay, assuming *Chlamydomonas* migrate from one edge to the other edge of the 100 mm Petri dish, the migration speed during chemotaxis was found to be roughly 0.6–1.2 µm s^−1^. These observations indicate that the cellular migration during ammonium chemotaxis is much slower than during phototaxis. This finding supports the idea that the steering mechanism of ammonium chemotaxis differ from that of phototaxis.

### Effects of light, circadian rhythm, and trophic conditions on *Chlamydomonas* ammonium chemotaxis in the Petri dish assay

We found that *Chlamydomonas* migrates towards ammonium in the presence and absence of homogeneous light; however, the migration pattern towards the source ammonium was not as clear without continuous light exposure (Fig. 2, Supplementary Fig. S9). In our conditions, the accumulation and dispersion of the cells around the source and from the sink agarose blocks, respectively, occurred most consistently in experiments under homogeneous light exposure. *Chlamydomonas* encodes at least eight light receptors (opsin, channelrhodopsin, histidine-kinase rhodopsin, phototropin, cryptochrome, UV resistance locus 8, cytochrome, plastocyanin) besides ones used in the photosynthetic machinery in the chloroplast^34–36^. Previous studies suggest that phototropin and cytochrome are involved in the mating of gametes and germination from zygote in *Chlamydomonas*^37,38^. On the other hand, studies suggest that rhodopsins are involved in phototaxis in *Chlamydomonas*^39,40^. An additional study suggested that phototropin activation suppresses nitrite chemotaxis during gamete formation in *Chlamydomonas*^41^. In our assay, *Chlamydomonas* was exposed to the ammonium gradient while in the vegetative phase. Although further analysis is required, our findings underpin the effect of light during *Chlamydomonas* ammonium chemotaxis.

We wished to identify a *Chlamydomonas* mutant with a defective ammonium chemotaxis response so that a genetic or molecular component could be revealed in the ammonium chemotaxis signal transduction pathway. However, all of the *Chlamydomonas* mutants tested in this study maintain a positive ammonium chemotaxis response (Supplemental Fig. S7). Our results suggest a few key points. First, the eyespot, the most upstream component in the signal transduction pathway in the phototaxis mechanism^4^, is not involved in the ammonium chemotaxis signal transduction. Second, the calcium-dependent shift in ciliate dominance required for phototaxis is not involved in the ammonium chemotaxis steering mechanism^6^. Lastly, ammonium uptake achieved by AMT4, the major ammonium transporter in *Chlamydomonas*^31^, is not involved in defining the cellular response against an ammonium gradient. This is distinct from the mechanisms governing bacterial collective migration in which nutrient uptake regulates the tactic response^14^. Further investigation is required to identify genetic components involved in the signal transduction, including the light and chemical receptors, in ammonium chemotaxis in *Chlamydomonas*.

We also examined the effect of circadian rhythm and trophic conditions on ammonium chemotaxis. In our culture conditions (12 h:12 h light:dark cycle in a medium containing acetate for 3–4 days before the assay), we did not detect a significant difference in the directed migration between the samples in the daytime (light cycle) or nighttime (dark cycle) (Supplementary Fig. S5). Conversely, when we used the samples cultured in an autotrophic condition (12 h:12 h light:dark cycle in a medium without acetate for 7–8 days before the assay), we did not detect an obvious migratory response towards the ammonium source (Supplementary Fig. S6). This suggests that the cells may alternate the sensitivity against an ammonium gradient based on the trophic conditions in the culture. A previously conducted capillary tube assay found that *Chlamydomonas* ammonium chemotaxis was most active during the nighttime and least active during the daytime. In this study, the cells were cultured autotrophically before the assay, and it was conducted without light exposure^7^. We suspect that the disagreement between the previously published study and our findings is due to several differences in experimental conditions, which include the trophic conditions of the culture before the assay, light exposure during the assay, and consideration of collective migration.

### *Chlamydomonas* collectively migrate during ammonium chemotaxis

*Chlamydomonas* exhibit collective migration during ammonium chemotaxis but not during phototaxis (Figs. 3, 4). In chemotaxis, individual cells first migrate toward the center of the population, suggesting *Chlamydomonas* may utilize the mechanism used in mammalian cells in which specialized molecules maintain cluster cohesion^17^. Our microscopic observation found an aggregation of cells (Supplementary Movie S2). The movement of the cilia in this aggregate was not clearly observed. This suggests that the physical interaction of the cilium-cilium or cilium-cell may maintain cluster cohesion. *Chlamydomonas* forms an aggregate, known as a palmelloid, when the cells are exposed to stressors^42–44^. However, we believe the aggregate observed during chemotaxis is not a palmelloid because a palmelloid forms a membrane around the aggregate (Supplemental Fig. S10), which we do not observe in the chemotactic aggregate (Supplementary Movie S2). The microscopic observation suggests that the aggregate formed during chemotaxis moves toward a source by non-aggregated individual cells. These non-aggregated cells essentially push the aggregate toward the source (Supplementary Movie S2). Although more analysis is required to understand the underlying mechanism, our findings revealed collective migration and the difference between phototaxis and chemotaxis in *Chlamydomonas* for the first time.

### AGG1 suppresses collective migration in *Chlamydomonas* while light promotes it during ammonium chemotaxis

**Figure 6:**
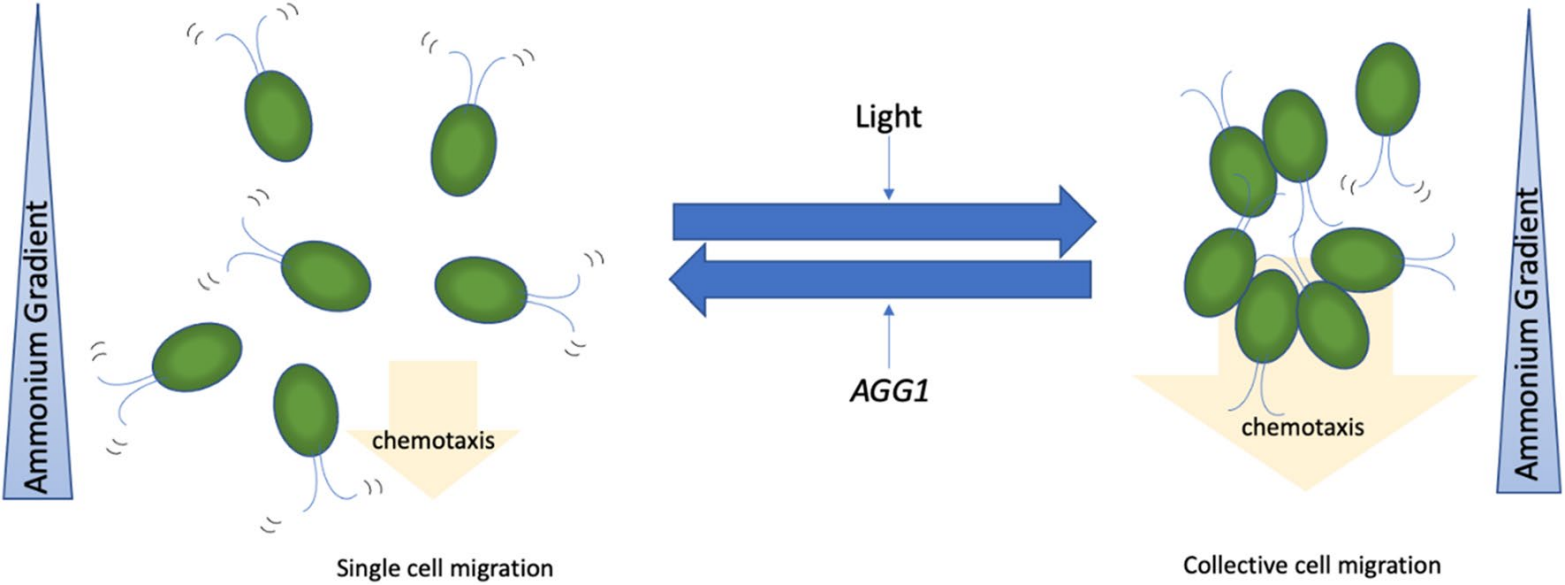
A proposed mechanism that ammonium chemotaxis is driven by collective migration in *Chlamydomonas*. A scheme showing how ammonium chemotaxis is driven by collective migration regulated by light exposure and AGG1. Blue triangles represent an increasing ammonium chemical gradient from top to bottom. Orange arrows indicate the direction and driving force of positive chemotaxis. Cells on the left show single cell migration, while cells on the right show collective cell migration.

CC-124, which carries an *agg1*^*−*^ mutation, showed clearer collective migration than CC-4533 (*AGG1*) when the Petri dish assay was performed at a low cell density (10^5^ cells/ml) (Fig. 4). The low density of the CC-124 cells was shown to respond to the ammonium gradient in the dark, while the low-density CC-4533 cells did not (Supplemental Fig. S9). The *agg1*^*−*^ mutant was initially isolated as a phototactic strain that exhibits negative phototaxis behavior^45^. Since then, the product of the *AGG1* gene has been considered a component in the phototaxis signaling pathway. Genetic analysis found that the *agg1*^*−*^ mutant in CC-124 abolishes expression of the AGG1 protein due to the insertion of a transposon in the gene^7^. The AGG1 protein contains two domains, a Fibronectin type III domain and a CHORD-Sgt1 (CS) domain. While the function of the Fibronectin type III domain is unclear, that of the CS domain is related to nuclear migration in animal cells^7^. The AGG1 protein is localized in the mitochondria in *Chlamydomonas*^7^. Because the molecular function of AGG1 is unknown, it is difficult to address how the different phenotypes of CC-124 observed during phototaxis and chemotaxis are linked at this point. This current work found that CC-124 expressing the recombinant AGG1 protein exhibited suppressed collective migration (Fig. 5, Supplementary Movie S3). This led to the hypothesis that AGG1 may function as a suppressor of collective migration during ammonium chemotaxis (Fig. 6). Light exposure made *Chlamydomonas* migrate more collectively during ammonium chemotaxis regardless of the *AGG1* gene status in the genome (Figs. 2, 4, Supplementary Fig. S9). Based on the findings in this study, we propose that collective migration is the major driving force of ammonium chemotaxis in *Chlamydomona*s (Fig. 6). Collective migration is promoted by light and suppressed by the activity of the AGG1 protein.

Previous studies indicate that light exposure enhances cilia-mediated adhesion in *Chlamydomonas*^46^, suggesting that adhesion may be involved in collective migration. Together with our finding that ammonium uptake does not affect tactic behavior, we speculate that the mechanisms governing collective migration in *Chlamydomonas* are more similar to mammalian cells that utilize the cell adhesion to control the collective migration than to bacteria that utilize nutrient uptake for control. Further analyses, including genetic analysis and single-cell tracking during chemotaxis with a steady-state ammonium gradient, will help to address whether these hypotheses are supported.

### Colonization and bet-hedging in ammonium chemotaxis

In this study, we found that *Chlamydomonas* collectively migrate towards ammonium under homogeneous light exposure, which is suppressed by the AGG1 protein. The advantage of collectively migrating is thought to promote directional migration, helping organisms colonize in their environment faster than if they migrate as single cells^14^. This could explain why *Chlamydomonas* collectively migrate towards ammonium under homogeneous light exposure because *Chlamydomonas* can photosynthesize, securing the fixing of the primary molecule, carbon, for their fitness. *Chlamydomonas* would then seek rapid colonization in an environment where the primary nutrient, nitrogen, is enriched. Without light exposure, bet-hedging strategies, in which the individuals stochastically express maladapted phenotypes (i.e., a cell migrates towards a poorer environment), may be dominated just in case environmental conditions at the source worsen suddenly or light is exposed from other directions^47,48^. AGG1 may function as a safeguard to maintain single-cell migration for the bet-hedging strategy under light exposure conditions.

## Methods

### *Chlamydomonas* strains

*Chlamydomonas* strains used in this study were sourced from the Chlamydomonas Resource Center (http://www.chlamycollection.org/). Cells were grown in 100 ml of Tris–acetate-phosphate (TAP) medium^7^ in a 250 ml flask stoppered with a sponge plug under a 12 h:12 h (light:dark, light intensity is ~ 30 μmol photo^ns^·m^−2^·s^−1^) cycle at 22 °C on a continuous rotary shaker (110 rpm). The transgenic CC-124 was generated as described previously^7^. When autotrophic conditions were required, the cells were cultured in a minimal medium that did not contain acetate in the TAP medium.

### Western blotting and phototaxis assay to confirm the expression of AGG1-3xHA protein

Expression of AGG1-3xHA (Human influenza hemagglutinin) was confirmed by western blotting using an anti-HA antibody and Wakabayashi’s phototaxis assay to confirm rescue from negative phototaxis phenotype as described in^7^.

### Petri dish assay

The Petri dish assay for chemotaxis with homogeneous light was performed in a photo box (FotoPro LED 20 × 20 Studio-in-a-Box, Fotodiox Inc.) with a reflective Mylar film (Mylar Diamond Film, TEXALAN) covering all sides. The Petri dish assay for chemotaxis in the dark was performed in incubators without light. The Petri dish assay for phototaxis was performed in the same photo boxes. Cultures in their nighttime (3–5 h into the dark cycle) were used for all assays 3–4 days after inoculation in the 100 ml culture described above. The cultures were placed in 50 ml centrifuge tubes and centrifuged for 5 min at 3000 rpm. Cells were washed with and resuspended in a TAP medium that did not contain ammonium (TAP-N). The cell number was counted with a hemacytometer after paralyzing the cilia movement by adding 300 mM KHPO_4_ at a 1:1 ratio. The cell density was adjusted to 10^6^/ml with the TAP-N medium and used immediately. Ten milliliters of the cell suspension were added to each Petri dish (100 mm in diameter, 15 mm deep). A lid was placed on the Petri dish and removed when a photo was taken. For chemotaxis experiments, agarose blocks (20 mm × 20 mm × 2 mm with 1.5% wt. agarose) were made with TAP-N that contained either 0 mM NH_4_Cl or 21 mM NH_4_Cl. The saturated agarose blocks were stored at 4 °C until use. A chemical gradient was introduced in the Petri dish by placing two agarose blocks containing either 0 mM or 21 mM NH_4_Cl on the opposite sides of the dish. For phototaxis experiments, Petri dishes were covered with a cardboard rectangle (125 mm × 125 mm × 45 mm) with one side open to the light source (Supplementary Fig. S8). All homogeneous light exposure was performed using a white LED light (~ 30 μmol photons·m^−2^ s^−1^) continuously exposed for the entire duration of the 24 h assay.

### Determination of chemotaxis Index

Photos of Petri dishes were taken at the 0, 3, 6, 12, and 24 h time points. The photos were analyzed by Fiji^49^. The photos were converted to 8-bit gray-scale images and inverted. The intensity around the agarose blocks was measured. Typically, an ROI (region of interest) was manually drawn so that the ROI was placed about 1 cm around a source agarose block (containing 21 mM NH_4_Cl) within a Petri dish. The same ROI was copied and used for a sink agarose block (containing 0 mM NH_4_Cl). The ROI was also copied and used in images taken at different time points so that the region where the intensity was measured was consistent during the time course. Background signals were obtained by measuring the intensity outside the Petri dish. These intensity values were imported into Excel (Microsoft Corporation) to calculate the chemotaxis index (CI). The CI was defined as:

2 $$CI = \left[ {\frac{{{\text{Average}}\;{\text{intensity}}\;{\text{around}}\;{\text{source }}}}{{{\text{Average}}\;{\text{intesity}}\;{\text{around}}\;{\text{sink }}}}} \right]$$

### Determination of doubling time

The doubling time of the cells was determined by counting cell numbers in Petri dishes placed in identical conditions to the chemotaxis assay, except that no agarose blocks were placed in them. The medium in the Petri dishes was TAP. The cells in the Petri dishes were collected at times 0, 6, 12, and 24 h. The equation $${A}_{t}={A}_{0}{(e)}^{rt}$$ was used to curve fit the changes of the cell number over time, in which $${A}_{t}$$ is the cell number at time $$t$$, $${A}_{0}$$ is the cell number at time $$0$$, and $$r$$ is a growth rate. The doubling time ($${t}_{d})$$ is defined as:

3 $${t}_{d}=\frac{\mathrm{ln}(2)}{r}$$

### Experimental evaluation of ammonium diffusion with a pH indicator in a Petri dish

Agarose blocks (1.5 wt.%) containing either 0 or 21 mM NH_4_Cl were placed in a dish with 10 ml of bromophenol blue solution (15 µM, pH 4.0). Images were taken every 5 min for 24 h.

### Computational modeling of mass transfer in a Petri dish

Diffusion of 600 µM bromophenol blue (pH is not adjusted) from a 1.5 wt.% agarose block (20 mm × 20 mm × 2 mm) into the water in a dish was quantified by taking images and analyzed by Fiji. This was accomplished by drawing a straight line from the edge of the source agarose to the bottom of the dish (Fig. S2A, 1 h), and the color intensity profile was plotted. The measured intensity was directly correlated to the concentration of dye diffused through the water. Using the known dimensions of the agarose block inside the dish, the measurement scale was defined for every image to convert between pixels in the image and the distance (in cm) in the dish. Images from 1, 4, 8, and 24 h time points were used for the analysis to approximate the diffusion coefficient of bromophenol blue. The mass transfer of chemicals is described by Fick’s law as follows as previously characterized by Sung et al.^24^:

S1 $$\frac{\partial c}{\partial t}=u\nabla c+D{\nabla }^{2}c$$

where C is the concentration of the dye (µM), u is the velocity of the fluid responsible for the convective mass transfer and is equal to zero in the case of this experiment (cm/s), and D is the diffusion coefficient of the molecules in the solution (cm^2^/s). The concentration gradient present in the dish can be predicted using a solution of Eq. S1 using the error function below:

S2 $$C\left(x, t\right)={C}_{0}\left[1-\mathrm{erf}\left(\frac{x}{2\sqrt{Dt}}\right)\right]$$

where C_0_ is the initial (source) concentration (µM), x (cm) is the distance, and t (s) represents time. A custom Python code was used to fit the experimental line scan data (Fig. S2A) to Eq. S2; however, since the collected data is measurements of the dye absorbance intensity, concentrations (C and C_0_) in Eq. S2 were replaced with intensity (I and I_0_). This is a common approach used to model mass transfer of dyes in water^50^. I_0_ was assumed equal to the highest measured intensity in the dish which corresponds to position immediately outside of the agarose block at the 1 h time point. Diffusion coefficients were found for the four timepoints and the average value was used to model the diffusion of bromophenol blue. This diffusion coefficient was approximated to be 1.42 × 10^−4^ (cm^2^/s), which showed an accurate alignment between the experimental and simulated data and validated that Equation S2 was a good fit for the mass transfer in the Petri dish (Fig. S2B). The approximated diffusion coefficient was used to model the diffusivity of bromophenol blue through the dish at all experimental time points (Fig. S2C). The trends and gradients predicted by the model accurately match what was observed in the experimental data (Fig. S2A), confirming the diffusion of bromophenol blue released from the agarose block through the water in the Petri dish. The diffusion coefficient predicted by the experimental data does deviate from the published diffusion coefficient of bromophenol blue in water (4.4 × 10^−6^)^51^. This difference can be attributed to the image processing and analysis approach. Since the experimental measurements are of absorbance intensity, and the collected data is being fit to a model originally designated for concentration values, it can be a factor in the over-approximation. Moreover, it is known that although the dye concentration and the corresponding absorbance have a linear correlation as described by the Beer–Lambert law, intensity and absorbance are described through an exponential correlation. As such, high concentrations of the dye being used to visualize the mass transfer resulted in greater light intensities when compared to the actual concentration of the dye itself, resulting in the observed variation in the diffusion coefficient.

### Microscopy observation of chemotaxis and phototaxis

Videos of the Petri dishes were captured at 10 h post-setup for chemotaxis. A Petri dish was placed on the stage of a LEICA inverted microscope (DMI6000 B). Videos were captured at 30 fps with an objective of 10×/N.A. 0.25 for 10 min.

### Video observation of chemotaxis in the transgenic strains

Images were captured every 5 min for 24 h to create a time-lapse video.

## Supplementary Information

Supplementary Information.

Supplementary Information.

## Data Availability

All data generated or analyzed during this study are included in this published article and the Supplementary Information.
