# Supplementary material for: Cells collectively migrate during ammonium chemotaxis in Chlamydomonas reinhardtii
Source: Sci Rep. 2023 Jul 4;13:10781. doi: 10.1038/s41598-023-36818-6 (PMC10319813; doi:10.1038/s41598-023-36818-6)
Supplement: Supplementary file 1 — Supplementary Information. [file 41598_2023_36818_MOESM1_ESM.pdf]

# Supplementary Information

**Supplementary Table S1** *Chlamydomonas* strains used in this study

| Strain Name | Description | Transgene/Genotype | Reference |
|---|---|---|---|
| #6 | Transgenic CC-124 | agg1p::AGG1-3xHA | 1 |
| #13 | Transgenic CC-124 | agg1p::AGG1-3xHA | 1 |
| CC-124 | Wild type | *nit1 nit2 agg1* $mt^{-}$ | 2 |
| CC-125 | Wild type | *nit1 nit2* $mt^{+}$ | 2 |
| *ptx1* (CC-2894) | Defected phototaxis (flagellar dominance mutant) | *ptx1* $mt^{+}$ | 3 |
| $amt4^{-}$ (CC-4042) | Defected ammonium accumulation (ammonium transporter mutant) | *amt4* $mt^{+}$ | 4 |
| *eye3-2* (CC-4316) | Defected phototaxis (eyespot-less mutant) | *eye3-2::NIT1* $mt^{+}$ | 5 |
| CC-4533 | Wild type (CLiP background strain) | *cw15* $mt^{-}$ | 6 |
| *pf14* (CC-613) | Paralyzed mutant (radial spoke-defect) | *Pf14* $mt^{-}$ | 7 |
| *pf18* (CC-1036) | Paralyzed mutant (central pair-defect) | *Pf18* $mt^{+}$ | 8,9 |

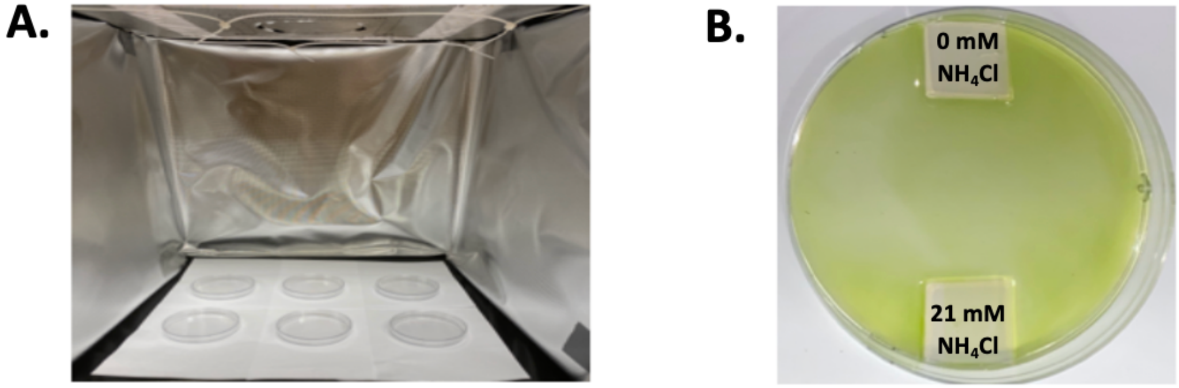


**Figure S1. Experimental setup of the Petri dish assay used to study ammonium chemotaxis in *Chlamydomonas***
(**A.**) A photo box is used for the assay to expose light to the top of the Petri dishes homogenously. (**B.**) After algae are placed into a 100 mm diameter Petri dish homogenously, a chemical gradient is introduced by placing 20 mm x 20 mm x 2 mm squares of 1.5 wt.% agarose containing either 0 mM of $NH_4Cl$ or 21 mM $NH_4Cl$.

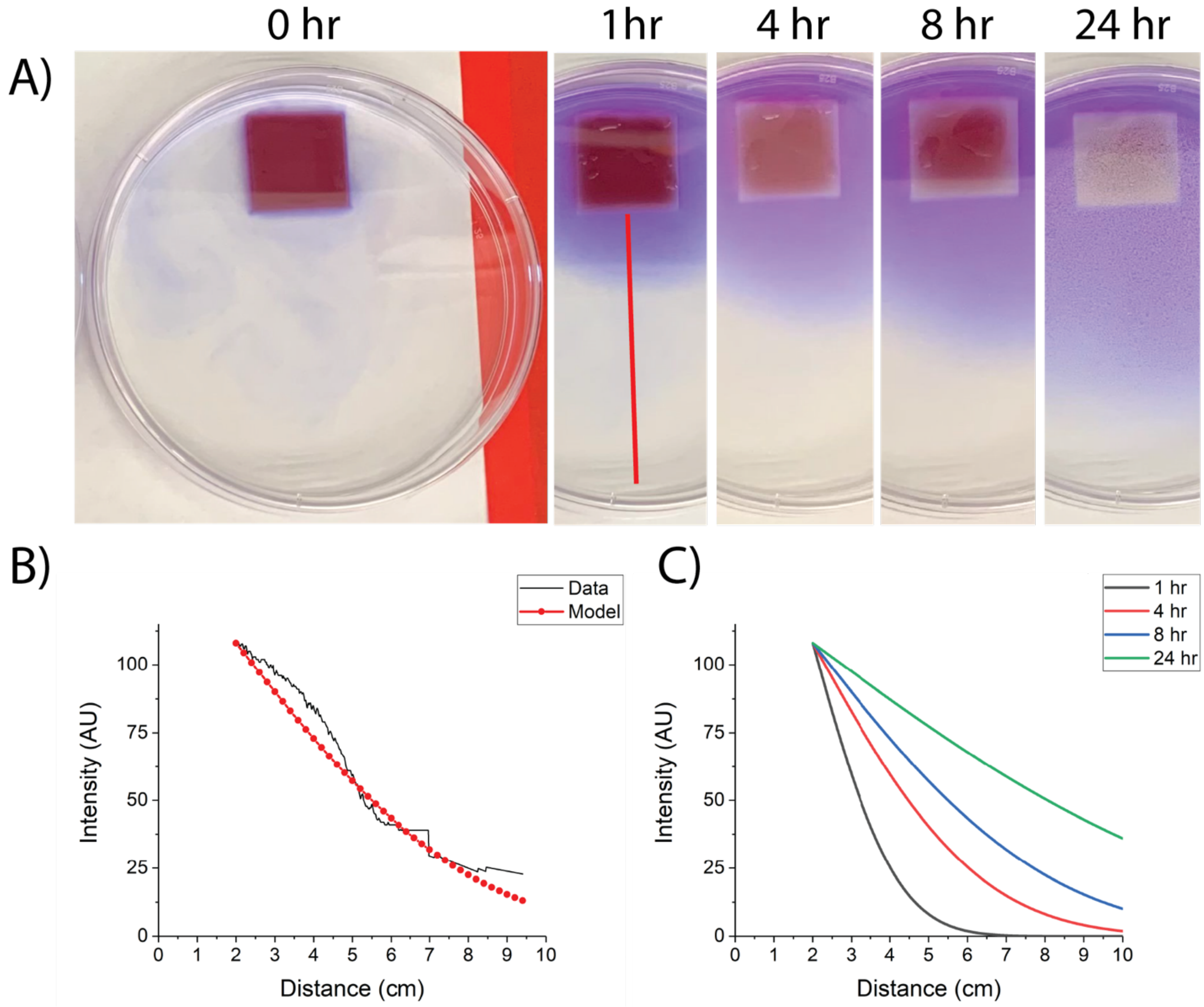


**Figure S2. Experimental and computational modeling of bromophenol blue mass transfer in the Petri dish assay**. A 20 mm x 20 mm x 2 mm block of agarose with 600 µM bromophenol blue (pH is not adjusted) was positioned on the top of a Petri dish. (**A.**) The dish was left at room temperature for 24 hours, with photos taken at 1, 4, 8, and 24 h. (**B.**) Experimental (solid black line) and simulated (red points and line) data for the 8 h time point was plotted, demonstrating the validity of the mass transfer model. (**C.**) Modeled time-dependent mass transfer across the dish resulting in a chemical gradient using an approximated diffusion coefficient from the experimental data and Fick's mass transfer model.

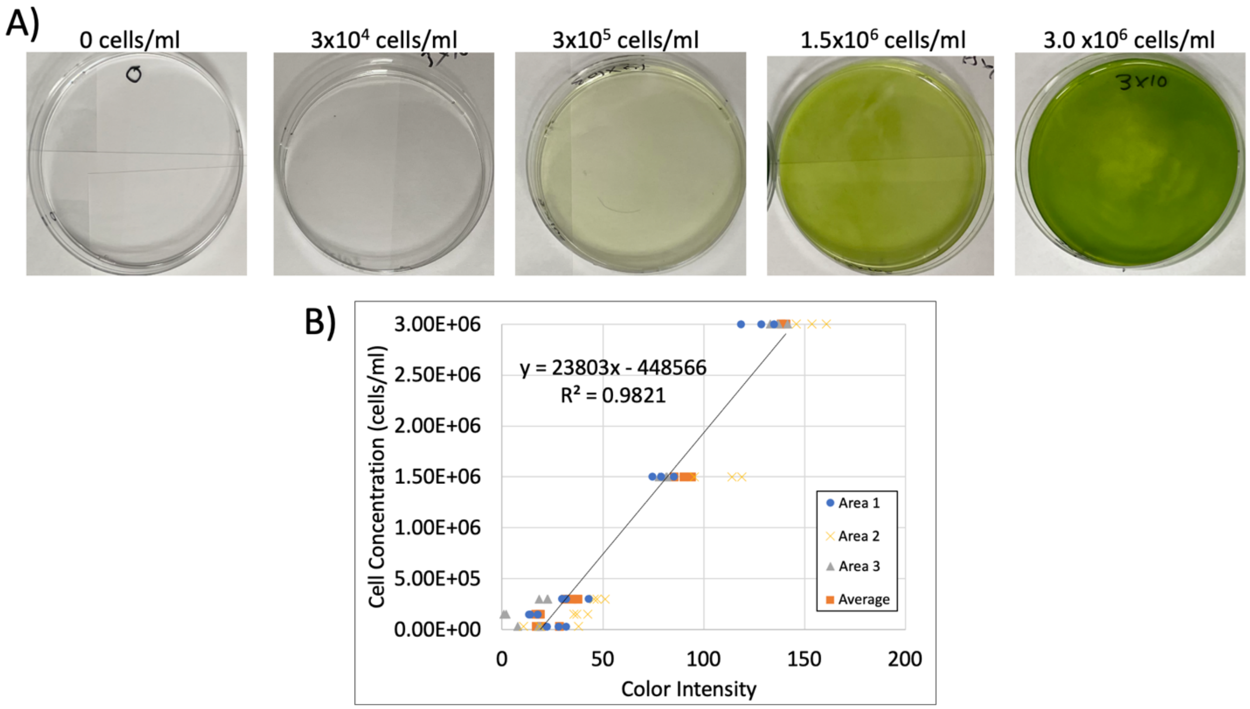


**Figure S3. A linear correlation was observed between cell concentration and color intensity in the Petri dish to allow for quantifying ammonium chemotaxis.**

(**A.**) Images of Petri dishes containing *Chlamydomonas* in different cell densities (concentrations).

(**B.**) Each data point with a circle, cross, or triangle showed intensity in three different areas randomly selected in the Petri dish. The squares represent the average intensity of those three areas. The graph shows a linear trend-line of the nine measurements at each concentration (three areas for three independent experiments).

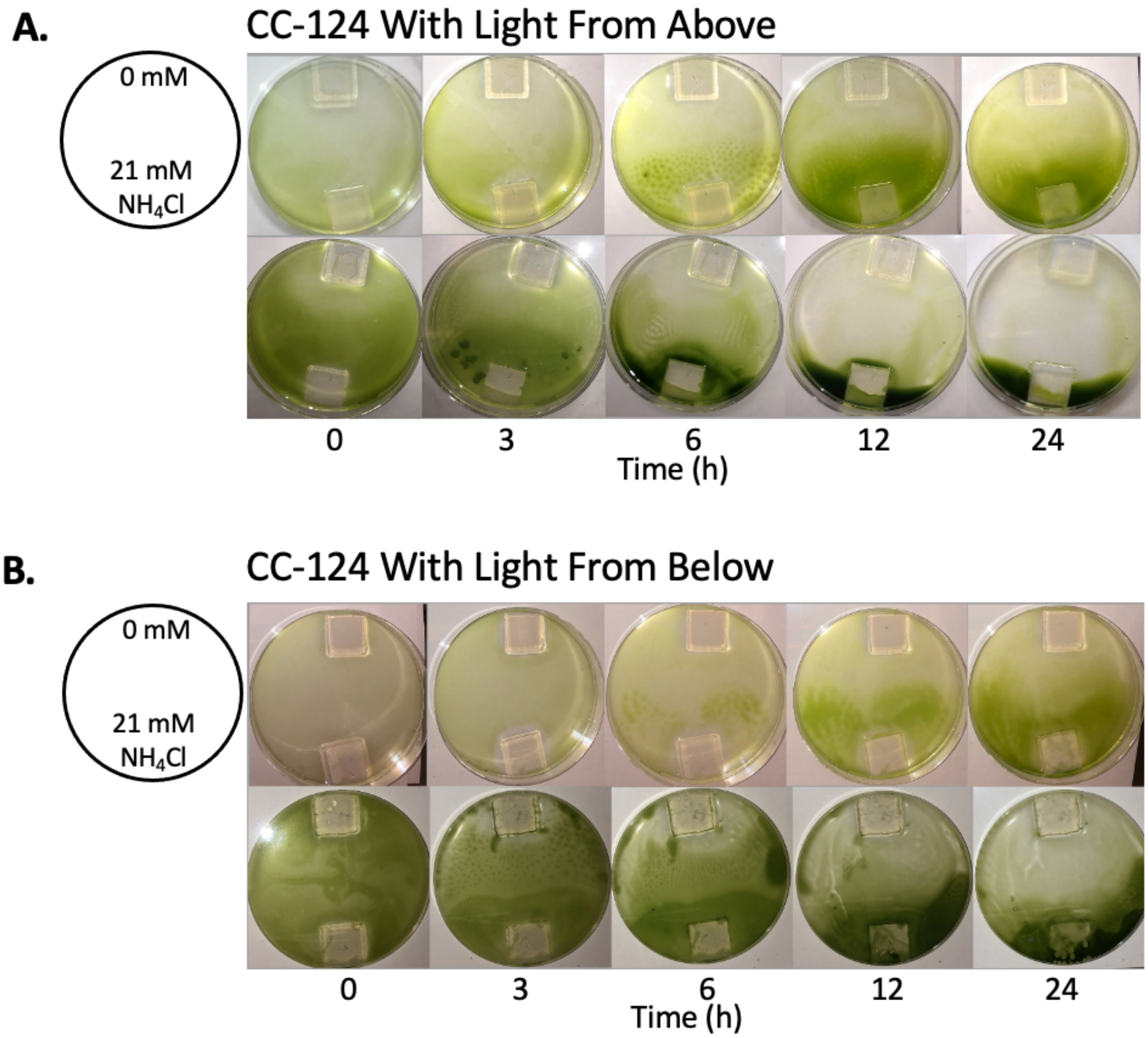


**Supplementary Figure S4: *Chlamydomonas* moves by swimming, not by gliding, during chemotaxis**

*Chlamydomonas* CC-124 (negative phototaxis strain) was placed in a Petri dish filled with TAP – N and exposed to light either from above (50 cm) or below (50 cm) the Petri dishes. The exposure from above induces the algae to accumulate on the bottom of the Petri dish, away from the light source, due to negative phototaxis. The light exposure from below induces the algae to accumulate on the surface of the medium, away from the light below, due to negative phototaxis. Photos were taken at the 0, 3, 6, 12, and 24 h time points. The two rows in each image montage represent two independent experiments. (**A.**) CC-124 exposed to light from above. (**B.**) CC-124 exposed to light from below. These findings suggest that the direction of light (from above or below) does not bias ammonium chemotaxis, indicating that CC-124 migrates by swimming in the solution and not by gliding on the surface of the dish.

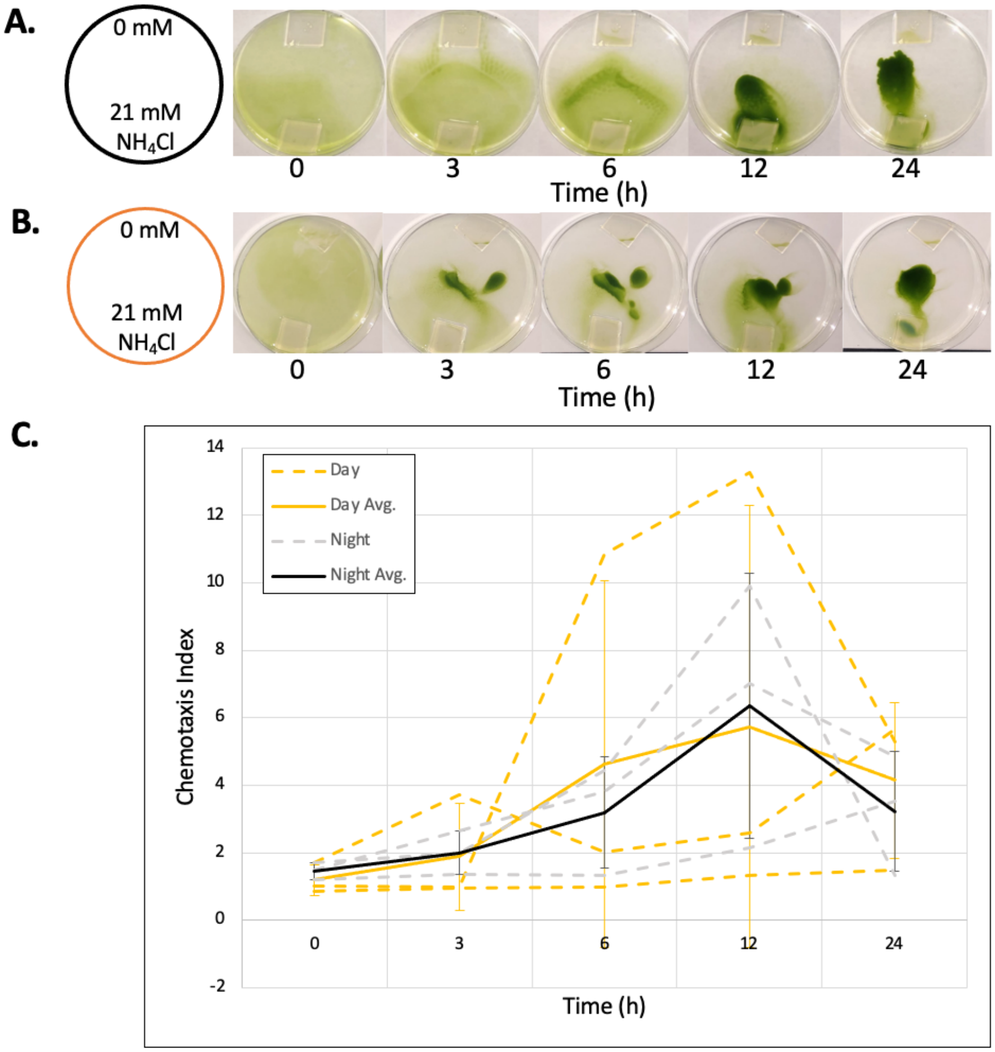


**Supplementary Figure S5. Ammonium chemotaxis of *Chlamydomonas* was observed during both the daytime (light cycle) and nighttime (dark cycle)**

*Chlamydomonas* CC-124 was grown in a 12h:12h (light:dark) cycle followed by performing a chemotaxis assay during either their nighttime (dark cycle) **(A)** or daytime (light cycle) **(B)** Cells were exposed to an ammonium gradient under homogenous light. The chemical gradient started from 21 mM $NH_4Cl$ at the bottom of the Petri dish to 0 mM $NH_4Cl$ at the top of the Petri dish in the photos. After setup, the photos were taken at 0, 3, 6, 12, and 24 h time points. **(C)** Orange and grey dotted lines connect the chemotaxis index (CI) values at each time point. The results of three independent experiments in the daytime (orange dotted line, Day) and nighttime (grey dotted line, Night) were shown, respectively. The solid orange line connects the average CI value in the daytime (Day Avg.) at each time point of the three independent experiments. The dark grey solid line connects the average CI values in the night (Night Avg.) at each time point of the three independent experiments. Error bars at each time point indicate the standard deviation of the three experiments.

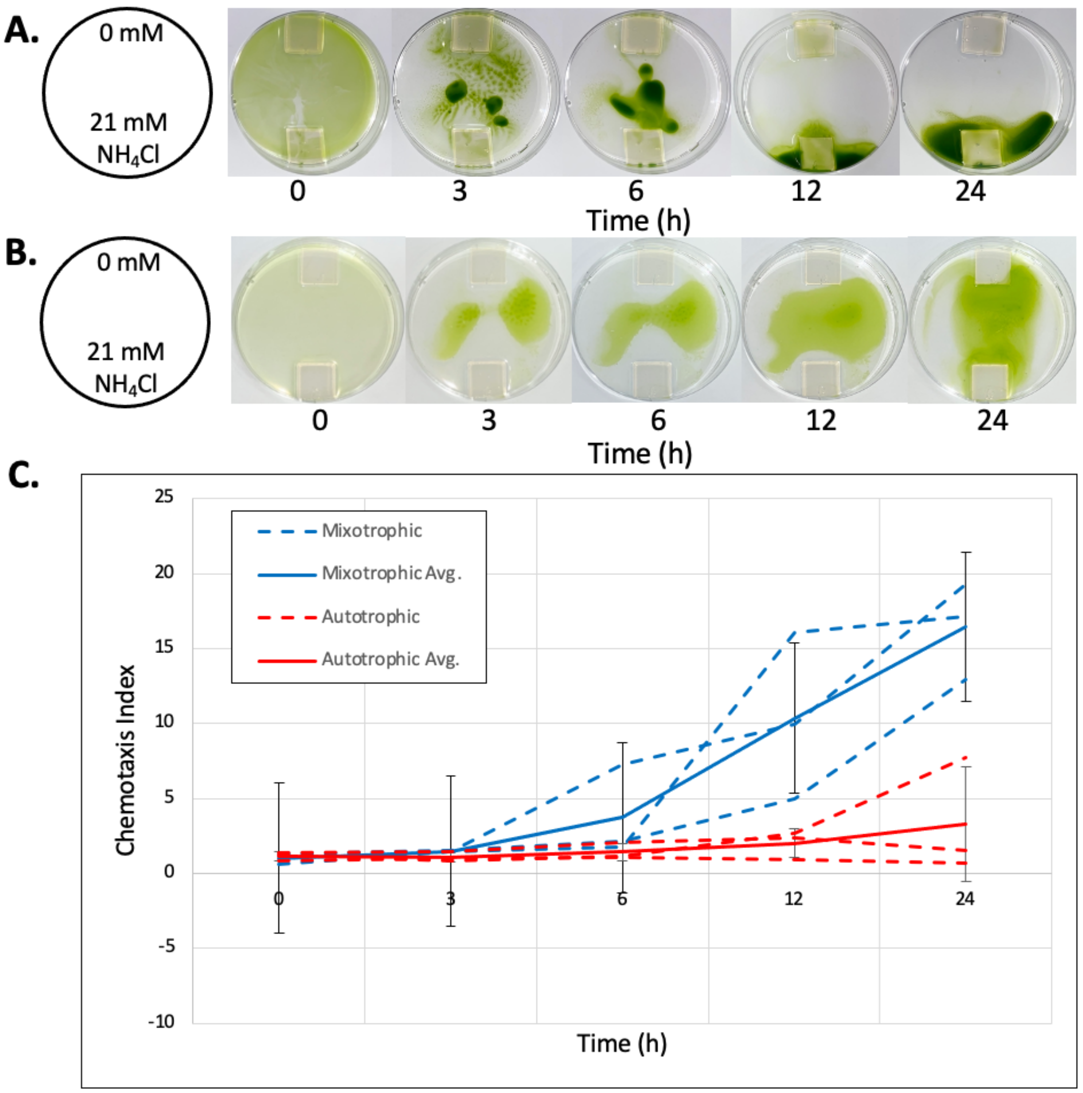


**Supplementary Figure S6. Ammonium chemotaxis of *Chlamydomonas* rarely occurs when the cells are cultured autotrophically and assayed in a medium not containing acetate**
*Chlamydomonas* CC-124 cells cultured in mixotrophic **(A)** or autotrophic **(B)** conditions were exposed to an ammonium gradient under homogenous light in the Petri dish assay. The chemical gradient started from 21 mM $NH_4Cl$ at the bottom of the Petri dish to 0 mM $NH_4Cl$ at the top of the Petri dish in the photos. After setup, the photos were taken at 0, 3, 6, 12, and 24 h time points. **(C)** Blue and red dotted lines connect the chemotaxis index (CI) values at each time point. The results of three independent experiments in mixotrophic (blue dotted line, Mixotrophic) and autotrophic (red dotted line, Autotrophic) were shown, respectively. The solid blue line connects the average CI value in the mixotrophic conditions (Mixotrophic Avg.) at each time point of the three independent experiments. The solid red line connects the average CI values in the autotrophic conditions (Autotrophic Avg.) at each time point of the three independent experiments. Error bars at each time point indicate the standard deviation of the three experiments.

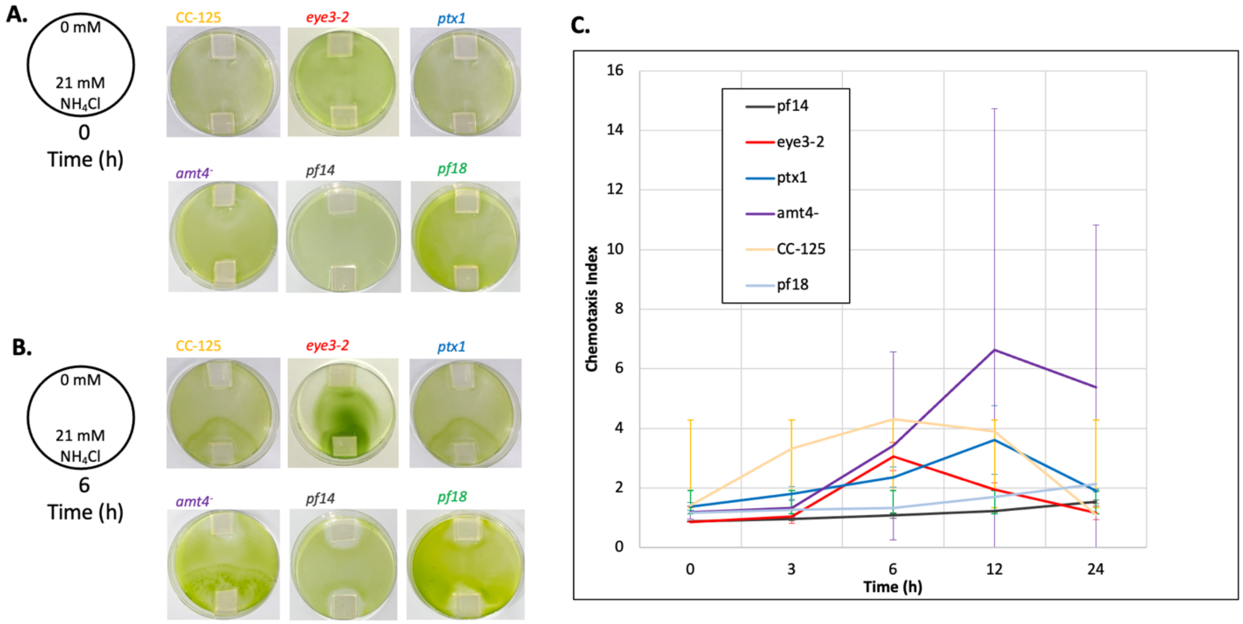


**Supplementary Figure S7. Chemotactic behavior in mutant strains**
***Chlamydomonas* mutant strains** *pf14* (CC-613), *pf18* (CC-1036), *eye3-2* (CC-4316), *ptx1* (CC-2894), and *amt4*$^{-}$ (CC-4042) were subjected to the ammonium chemotaxis assay together with a wild type strain CC-125 under homogeneous light exposure. **(A)** Representative photos of each strain soon after the samples were set (the 0 h time point). **(B)** Representative photos of each strain six hours after the samples were set. **(C)** The chemotactic index (CI) of each cell population was analyzed for each strain. The lines connect the average CI values at each time point of the three independent experiments. Error bars at each time point indicate the standard deviation of the three experiments. Notice *eye3-2*, *ptx1*, and *amt4*$^{-}$ are capable of ammonium chemotaxis while *pf14* and *pf18* are not. Read the main text for the detailed explanation.

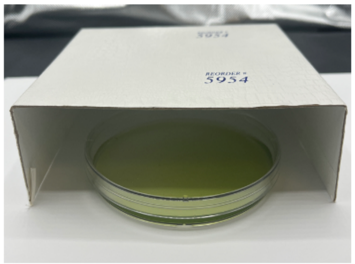

**Supplementary Figure S8. Phototaxis experimental setup**
After the algae were placed into the Petri dish without agar blocks in a photo box, a light gradient was established by covering a portion of the Petri dish with a cardboard box with an open side.

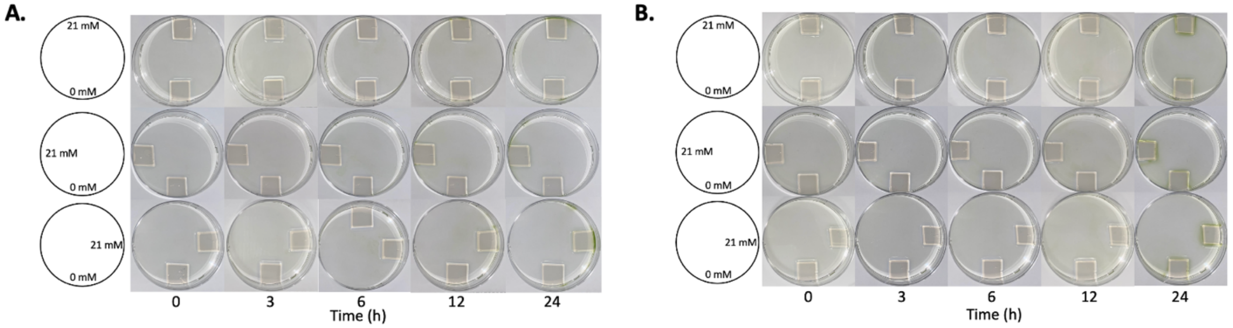


**Supplementary Figure S9. CC-124 (*agg1*⁻), but not CC-4533 (*AGG1*), exhibit positive chemotaxis at low cell densities in the dark**

Low cell densities ($10^5$ cells/ml) were exposed to chemical gradients from three different directions in the dark. The top row has a chemical gradient from 0 mM $NH_4Cl$ at the bottom of the Petri dish to 21 mM $NH_4Cl$ at the top of the Petri dish. The second row has a chemical gradient from 0 mM $NH_4Cl$ at the bottom of the Petri dish to 21 mM $NH_4Cl$ at the left of the Petri dish. The third row has a chemical gradient from 0 mM $NH_4Cl$ at the bottom of the Petri dish to 21 mM $NH_4Cl$ at the right of the Petri dish. Photos were taken at the 0, 3, 6, 12, and 24 h time points for either CC-124 **(A)** or CC-4533 **(B)** CC-124 cells were found to accumulate near the source agarose **(A)**, while the CC-4533 cells accumulated near both the source and sink agarose **(B)**, indicating no chemotactic response.

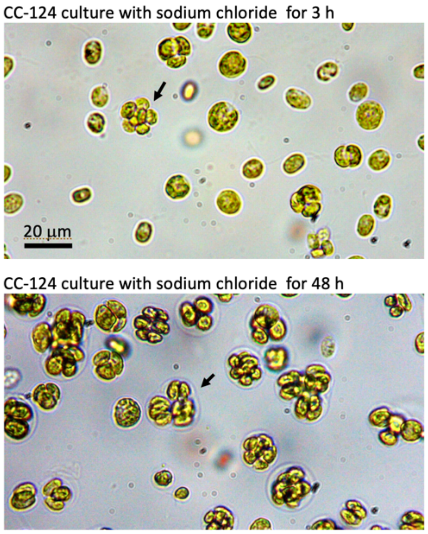


**Supplementary Figure S10. The form of palmelloids were different from that of aggregate occurring during ammonium chemotaxis.** A final concentration of 150 mM sodium chloride (NaCl) was added to three days-old CC-124 culture ($10^6$ cells/ml) and incubated to form palmelloids[10]. Images of the cultures were taken with a 40x objective lens at 3 h and 48 h after adding NaCl. Notice the aggregates were formed with about ten cells surrounded by a membrane (pointed out with a black arrow) at both 3 and 48 h. The form of the aggregates clearly differs from that during ammonium chemotaxis (Supplementary Movie S2).

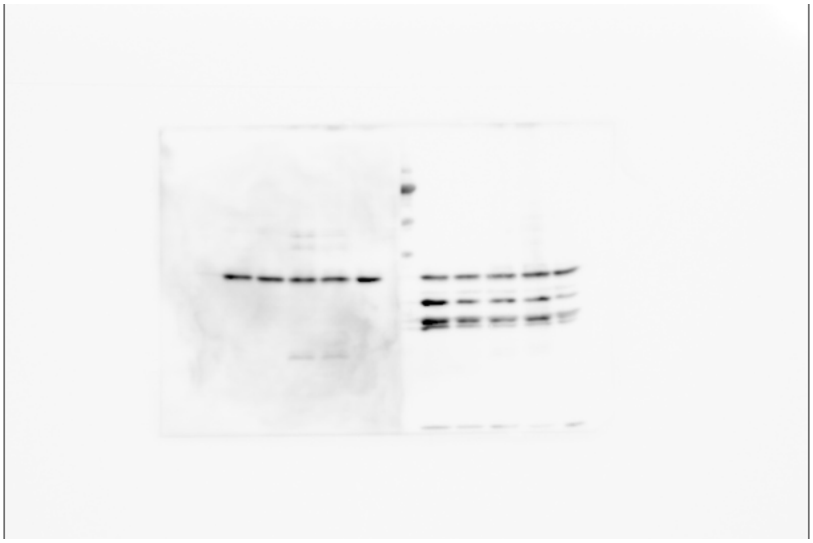

**Supplementary Figure S11. The original, unprocessed image of the blot in Figure 5**. Two blots were originally imaged side-by-side. Both blots comprise five samples (lanes). The blot on the right also contains one molecular-size marker (one additional lane). The blot on the left was reacted with an anti-HA antibody. The blot on the right was reacted with another (not anti-HA) antibody. In Figure 5 in the main text, the first to the fourth lane from the left on the left side blot was cropped and displayed.

**Supplementary Movie S1. Visualization of chemical diffusion and *Chlamydomonas* migration in the Petri dish assay**
**Left**: Chemical diffusion was observed from an agarose block containing 21 mM (at the bottom of the dish) or 0 mM (at the top of the dish) $NH_4Cl$ with 10 ml of a bromophenol blue solution (a colorimetric pH indicator) homogenously added to the dish. **Right**: *Chlamydomonas* migration was observed with an agarose block containing 21 mM $NH_4Cl$ placed inside a Petri dish (at the bottom of the dish) filled with 10 ml of CC-124 in TAP–N. An agarose block containing 0 mM $NH_4Cl$ was placed on the opposite side (at the top of the dish).

**Supplementary Movie S2. Microscopy observation of collective migration during chemotaxis**
A population of *Chlamydomonas* CC-124 was exposed to a chemical gradient from 0 mM $NH_4Cl$ at the left of the Petri dish to 21 mM $NH_4Cl$ at the right of the Petri dish. The movie was recorded at 30 fps for 10 min.

**Supplementary Movie S3. Transgenic strains of *Chlamydomonas* show reduced collective migration during chemotaxis.** CC-124, and the transgenic CC-124, #6, and #13 were exposed to ammonium chemical gradients under homogenous light exposure. A chemical gradient was formed in the dishes on the top row by placing an agarose block containing 0 and 21 mM $NH_4Cl$ on the left and right sides, respectively. No chemical gradient was established in dishes on the bottom row (an agarose block containing 0 mM $NH_4Cl$ was placed on both the right and left sides).

## References


1 Ide, T. *et al.* Identification of the agg1 mutation responsible for negative phototaxis in a "wild-type" strain of Chlamydomonas reinhardtii. *Biochem Biophys Rep* **7**, 379-385, doi:10.1016/j.bbrep.2016.07.016 (2016).

2 Gallaher, S. D., Fitz-Gibbon, S. T., Glaesener, A. G., Pellegrini, M. & Merchant, S. S. Chlamydomonas Genome Resource for Laboratory Strains Reveals a Mosaic of Sequence Variation, Identifies True Strain Histories, and Enables Strain-Specific Studies. *Plant Cell* **27**, 2335-2352, doi:10.1105/tpc.15.00508 (2015).

3 Horst, C. J. & Witman, G. B. ptx1, a nonphototactic mutant of Chlamydomonas, lacks control of flagellar dominance. *Journal of Cell Biology* **120**, 733-741, doi:10.1083/jcb.120.3.733 (1993).

4 Kim, K. S. *et al.* Spontaneous mutations in the ammonium transport gene AMT4 of Chlamydomonas reinhardtii. *Genetics* **170**, 631-644, doi:10.1534/genetics.105.041574 (2005).

5 Lamb, M. R., Dutcher, S. K., Worley, C. K. & Dieckmann, C. L. Eyespot-assembly mutants in Chlamydomonas reinhardtii. *Genetics* **153**, 721-729, doi:10.1093/genetics/153.2.721 (1999).

6 Li, X. *et al.* A genome-wide algal mutant library and functional screen identifies genes required for eukaryotic photosynthesis. *Nat Genet* **51**, 627-635, doi:10.1038/s41588-019-0370-6 (2019).

7 Luck, D., Piperno, G., Ramanis, Z. & Huang, B. Flagellar mutants of Chlamydomonas: studies of radial spoke-defective strains by dikaryon and revertant analysis. *Proc Natl Acad Sci U S A* **74**, 3456-3460, doi:10.1073/pnas.74.8.3456 (1977).

8 Adams, G. M., Huang, B., Piperno, G. & Luck, D. J. Central-pair microtubular complex of Chlamydomonas flagella: polypeptide composition as revealed by analysis of mutants. *Journal of Cell Biology* **91**, 69-76, doi:10.1083/jcb.91.1.69 (1981).

9 Bloodgood, R. A. & Salomonsky, N. L. Use of a novel Chlamydomonas mutant to demonstrate that flagellar glycoprotein movements are necessary for the expression of gliding motility. *Cell Motility* **13**, 1-8, doi:https://doi.org/10.1002/cm.970130102 (1989).

10 Khona, D. K. *et al.* Characterization of salt stress-induced palmelloids in the green alga, Chlamydomonas reinhardtii. *Algal Research* **16**, 434-448, doi:https://doi.org/10.1016/j.algal.2016.03.035 (2016).
